# Systemic Sclerosis and the Gastrointestinal Tract—Clinical Approach

**DOI:** 10.5041/RMMJ.10258

**Published:** 2016-10-31

**Authors:** Yolanda Braun-Moscovici, Rita Brun, Marius Braun

**Affiliations:** 1 B. Shine Rheumatology Unit, Rambam Health Care Campus, Haifa, Israel; 2 Rappaport Faculty of Medicine, Technion–Israel Institute of Technology, Haifa, Israel; 3 Department of Gastroenterology, Rambam Health Care Campus, Haifa, Israel; 4 Liver Institute, Beilinson Hospital, Petach Tiqwa, Israel; 5 Sackler School of Medicine, Tel Aviv University, Tel Aviv, Israel

**Keywords:** Gastric antral vascular ectasia, gastrointestinal tract involvement, systemic sclerosis

## Abstract

Systemic sclerosis (SSc) is a multisystem disease characterized by functional and structural abnormalities of small blood vessels, fibrosis of the skin and internal organs, immune system activation, and autoimmunity. The gastrointestinal tract is involved in nearly all patients and is a source of significant morbidity and even mortality. The aim of this review is to summarize the pathogenesis and to provide a clinical approach to these patients.

## INTRODUCTION

The first case of scleroderma was diagnosed by Hippocrates in the fourth century BC in a patient with “thick skin.”^1^ More than two thousand years later, in 1836, Giovambattista Fantonetti coined the term “scleroderma,” which is derived from Greek terminology “skleros” (hard) and “derma” (skin).^1^ The term “systemic sclerosis” was conceived by Robert H. Goetz, a heart surgeon, in 1945, who described scleroderma as a disease that infiltrates several internal organs.^2^

## PATHOGENESIS

Systemic sclerosis (SSc) is a multisystem disease characterized by functional and structural abnormalities of small blood vessels, fibrosis of the skin and internal organs, immune system activation, and autoimmunity. The cause of SSc is unknown. An integrated hypothesis of the pathogenesis of SSc includes a combination of abnormalities in the vascular and in the immune systems on a background of genetic susceptibility and in the presence of environmental stimuli, which leads to further augmentation of the immune system’s activation and, ultimately, to fibroblast proliferation, collagen deposition, and destruction of normal tissue architecture.^3^

The vascular hypothesis suggests that the primary event in SSc occurs at the level of capillaries and small vessels and manifests as endothelial cell injury and activation. Vascular pathology is characterized by abnormal vasoreactivity, dysregulation of vasoconstrictive molecules and their receptors, upregulation of intracellular signaling kinases, altered balance of hypoxia-induced vascular growth factors, and aberrant function of vascular cells and autoimmune effector cells, which all lead to insufficient neoangiogenesis.^4^–^10^

During the last decade, studies have emphasized the role of the innate and the adaptive immune system in the pathogenesis of SSc. Genome-wide approaches have revealed that increased expression of genes associated with SSc susceptibility and/or disease phenotype plays a major role in the regulation of the immune system. T cells, fibroblasts, growth factors, chemokines, and endothelin-1 are all key factors in disease pathophysiology.^11^–^16^

Systemic sclerosis has been classified according to the extent of clinically detectable skin tightness into limited cutaneous SSc (hardening confined to skin from elbows distally and from knees distally) and diffuse cutaneous SSc (hardening of skin including proximal extremities and the trunk).^17^ Both forms involve the internal organs.

Involvement of the gastrointestinal tract (GIT) in SSc is extremely frequent; it is a leading cause of morbidity and the third most common cause of mortality in this disease. Esophageal abnormalities occur in up to 90% of patients, stomach involvement can be documented in 50% or more of patients, and small bowel, colonic, and anorectal involvement occur in 50%–70% of SSc patients.^18^–^20^

The pathogenesis of GIT involvement is thought to include early vascular damage to the vasa nervorum of the nerves innervating the GIT. This leads to neurological dysfunction, particularly involving autonomic pathways.^21^,^22^ The activation of the immune system may contribute to neurological dysfunction by production of antibodies which specifically inhibit M3-muscarinic receptor-mediated enteric cholinergic neurotransmission.^23^ Endothelial/lymphocyte activation leads to prominent infiltration of CD4+ T lymphocytes as well as CD20+ B lymphocytes into the gastric mucosa of patients with SSc and perhaps represents an early event in gastrointestinal (GI) pathology.^24^ With damage to innervation, smooth muscle atrophies and is eventually replaced by fibrotic tissue. With increasing atrophy and tissue replacement, the GIT becomes progressively less effective and less responsive to therapeutic agents.^25^

## SYMPTOMS AND SIGNS

Motility disorders and vascular mucosal lesions are the main manifestations of GIT involvement in SSc. The entire GIT may be involved from the mouth to anus in both limited and diffuse SSc.

Oral cavity abnormalities are common in SSc. Tightening of the perioral skin secondary to fibrosis may cause severe feeding impairment. Xerostomia due to Sicca syndrome may occur in 14%–20.5% of SSc patients and may further decrease oral intake.^26^

Esophageal involvement is the most frequent gastrointestinal manifestation of SSc and occurs in up to 90% of patients. Multiple abnormalities of esophageal function cause the clinical manifestations of severe gastroesophageal reflux and dysphagia to liquids and solids. The hallmark of SSc in the esophageal body is ineffective esophageal motility with low or absent contractile activity. Subsequently, the lower esophageal sphincter (LES) is hypotensive, and hiatal hernia is common, resulting in almost free regurgitation of gastric acidic contents into the esophagus. In addition, saliva and esophageal mucosal secretions production is reduced. Heartburn and dysphagia are the most common complaints. Hoarseness, atypical chest pain, nocturnal cough, and regurgitation may also occur.

Ineffective motility, hypotensive LES, poor acid and bolus clearance, and lack of buffer secretions all contribute to esophageal mucosal damage secondary to refractory acid reflux. Late complications include esophageal stenosis, strictures, and, ultimately, Barrett’s esophagus and intestinal metaplasia.^19^ The prevalence of Barret’s esophagus in SSc was found to be 12.7%, similar to the prevalence in patients with gastroesophageal reflux disease (GERD).^27^ An increased risk of esophageal adenocarcinoma was reported in SSc patients and was associated with the occurrence of dysplasia in Barrett’s esophagus.^28^

Gastroesophageal reflux disease was suggested to be a risk factor for the development of interstitial lung disease.^29^

Gastroparesis is common in SSc patients, but its true prevalence is unknown. It is important to be aware of diagnosis and actively look for it, as its appropriate management can relieve the patient’s symptoms significantly.

Early satiety, postprandial fullness, nausea and vomiting, regurgitation of gastric contents, abdominal pain, and, in severe cases, malnutrition due to inability to maintain adequate oral intake are the clinical manifestation of gastroparesis.

Autonomic dysfunction plays an important role in the pathogenesis of this dysmotility.^21^

Small bowel involvement has been reported in 50%–70% of SSc patients^18^–^20^ and may lead to high morbidity and life-threatening complications, such as severe malabsorption and pseudo-obstruction. Small bowel hypomotility induces stasis of intestinal contents and small intestinal bacterial overgrowth (SIBO), which contribute to bloating, abdominal pain, nausea, vomiting, diarrhea, malabsorption, and weight loss.^30^ The prevalence of SIBO in SSc has been reported to be 30%–62%.^31^–^35^ Small intestinal bacterial overgrowth is one of the main pathogenetic factors of malabsorption which is associated with 50% mortality over 8.5 years in SSc patients.^36^ Rare or absent motor migratory complexes (MMCs), which serve as a house-keeping mechanism of intestines, contribute to SIBO, while decreased postprandial contractility of the small intestine is one of the causes of postprandial pain and discomfort. Clinically, diarrhea, bloating, and nutritional deficiencies due to malabsorption should raise the suspicion of SIBO.

Intestinal pseudo-obstruction is a rare cause of hospitalization in patients with SSc, but is associated with high in-hospital mortality.^37^

Pneumatosis cystoides intestinalis (PCI) is a rare complication of SSc and is considered a sign of poor prognosis.^38^,^39^ It is characterized by development of multiple intramural air-filled cysts, due to anaerobic bacterial overgrowth in the intestine and increased intraluminal hydrogen production. The cysts may rupture and cause pneumoperitoneum and secondary peritonitis. The risk of perforation is already increased in SSc patients due to fibrosis and loss of compliance of the intestinal wall.^40^

The colon is frequently involved in SSc, although it is not always symptomatic. Abnormal motility pattern has been found in 75% of asymptomatic SSc patients.^41^

Constipation and fecal incontinence due to reduced colonic motility and hypotensive anal sphincter are the main issues involving the colon.^42^ Fecal incontinence is an under-reported but frequent complication of SSc. Patients with diarrhea are especially prone to incontinence episodes.

Malnutrition and weight loss result from the multiple anatomic and functional abnormalities through the whole gastrointestinal tract in SSc, but studies assessing their prevalence are lacking.

Vascular lesions of the mucosa may cause severe anemia in SSc patients. The lesions may be scattered throughout the entire intestine or may involve only the stomach antrum (gastric antral vascular ectasia).^43^ Gastric antral vascular ectasia (GAVE) is characterized by a pathognomonic endoscopic pattern, mainly represented by red spots either organized in stripes radially originating at the pylorus (“watermelon stomach”) or arranged diffusely (“honeycomb stomach”).^44^ “Watermelon stomach” is the “classic” and more familiar form of GAVE. There are conflicting data regarding the prevalence of GAVE in SSc. Previous studies estimated a 1%–5.7% prevalence of GAVE in SSc patients.^45^,^46^ On the other hand, a study performed in patients with early diffuse SSc reported a much higher prevalence of GAVE: 22.3%.^47^ A recent study, using video capsule endoscopy (VCE), found evidence of “watermelon stomach” in 18% of SSc patients.^48^

## CLINICAL APPROACH AND ASSESSMENT TOOLS

In daily practice, the patient presents with a mixed clinical picture of refractory GERD, diarrhea, bloating, dysphagia, weight loss, and nutritional deficiencies. The diagnostic studies should be directed to identify the GI site involved, assess severity, and rule out other etiologies. Systematic evaluation of motoric function of the GI tract in SSc patients enables the clinician to build an appropriate treatment plan for the individual patient.

Anamnesis should be directed to identify the most bothersome symptoms of the patient. First, nutritional status should be evaluated, as often weight loss is a sensitive sign of poor functional status of the GI tract. Questions regarding symptoms of gastroparesis and fecal incontinence should be actively asked, because patients may have difficulties in sharing this information.

There is no single objective measure to assess the extent and severity of GI involvement in SSc patients.

Upper gastrointestinal (UGI) endoscopy is the gold standard for esophagus and stomach morphology assessment. The procedure is a means for visualizing tissue, for sampling, and for therapeutic interventions (e.g. in cases of bleeding from GAVE). Standard endoscopic imaging is useful for the detection of grossly visible lesions but may be less sensitive for the detection of early or subtle mucosal changes.

Gastroscopy is the gold standard for diagnosis of GAVE and for assessment of its severity. Gastric biopsy can help to diagnose the condition in equivocal cases. The histological pattern, although not pathognomonic, is characterized by the co-presence of ectasia and/or fibrin thrombi, spindle cell proliferation, and fibrohyalinosis. Gastric antral vascular ectasia can also be treated during UGI endoscopy using argon plasma coagulation.

In addition, information about the functional motility status of the upper GI tract can be obtained during UGI endoscopy: esophageal and gastric contents despite fasting, dilated esophagus, widely opened gastroesophageal junction, hiatal hernia, and lack of peristalsis are highly suggestive of hypomotility.

Evaluation of motor GI function should be performed in patients with symptoms suggesting motility abnormalities. High-resolution esophageal manometry is a new technique to evaluate esophageal motility. Reflux extent and severity, as well as a response to acid suppression medications, is studied using esophageal reflux monitoring with pH or pH/impedance probes based on intranasal catheter, or, recently, the more comfortable wireless Bravo pH-metry capsule.

Gastric emptying can be assessed by gastric scintigraphy or breath test.

Small intestinal bacterial overgrowth is diagnosed by breaths tests, which have multiple limitations, or, rarely performed clinically, by culture of jejunal aspirate.^49^ A more practical approach would be empirically treating SIBO with antibiotics and a retrospective diagnosis based on clinical response.^50^

Colonic transit time can be non-invasively measured using the SITZMARKS test (ingestion of a capsule containing 24 radiopaque markers that are visible throughout the digestive tract via X-ray).

Anorectal function is studied by anorectal manometry, preferably using high-resolution technology, which provides information of the functional status of the sphincter. Transrectal ultrasound (US) can be used to visualize the anatomy of internal and external anal sphincters. Magnetic resonance imaging has been used for evaluation of anorectal anatomy in SSc patients, but it is much more expensive and without clear advantages in this patient group compared to US.^51^

Video capsule endoscopy (VCE) identifies a high prevalence of gastrointestinal mucosal abnormalities, especially potentially bleeding vascular mucosal lesions (watermelon stomach, gastric and/or small intestinal telangiectasia, gastric and/or small intestinal angiodysplasia).^48^ Hypomotility problems in SSc may raise concern regarding the use of VCE in these patients.

## TREATMENT

To date, the management of GIT involvement in SSc remains empirical and symptom-driven. The ultimate goal of a systematic complex approach to GI abnormalities in scleroderma patient is the improvement of their nutritional status and quality of life.

High-dose, twice daily acid suppression treatment with a proton pump inhibitor is a mainstay therapy for GERD. Sometimes a combination with H2 blockers (ranitidine, famotidine) is needed to control the night-time breakthrough acid reflux. It is important to notice that poorly controlled GERD in SSc patients, despite optimal medical treatment, is often secondary to gastroparesis, and specific measures to improve gastric emptying should be incorporated into the treatment plan.

Treatment of gastroparesis starts with nutritional intervention—multiple small meals and low-fiber diet. When needed, pro-kinetic medications (domperidone, metoclopramide, erythromycin) are added to improve gastric emptying.

Small intestinal bacterial overgrowth is usually treated with antibiotics. Recently, rifaximin has been used for treating SIBO, with the advantages of being a non-absorbable antibiotic with few systemic side effects, as well as possible positive influences on intestinal flora.^52^ Octreotide has been reported to be helpful in selected cases, especially in patients with recurrent pseudo-obstruction of the small bowel.^41^,^53^

Fecal incontinence requires a complex approach, combining medical and nutritional interventions with physical therapy, preferably anorectal biofeedback training. Unfortunately, the response rate to these therapies is not usually satisfactory in SSc patients. New therapies for fecal incontinence, such as sacral nerve stimulation, have been reported to be unsuccessful in scleroderma.^54^

Nutritional deficiencies should be corrected (vitamins B12, D, etc.). Deficiency of vitamin B12 is common and should be treated. In case of low BMI an appropriate nutritional plan should be developed for each patient. Patients with chronic intestinal pseudo-obstruction who cannot tolerate enteral feeding may need prolonged total parenteral nutrition.^55^,^56^

Some studies suggest probiotics may be useful for treatment of SSc-associated distention and bloating, but the small number of patients and the diversity of probiotics used do not permit any consistent treatment recommendation.^57^

Gastric antral vascular ectasia should be treated by endotherapy—argon plasma coagulation (APC). Treatment with APC can reduce the need for blood transfusions.^46^ Most patients with early diffuse SSc and GAVE will need recurrent endoscopic coagulations to overcome UGI bleeding. In these patients with early diffuse progressive disease, concomitant immune suppression with cyclophosphamide or mycophenolate mofetil might contribute to significant improvement and eventually to final resolution of the UGI bleeding.^58^–^60^^ and our unpublished data^

Data regarding the influence of immunomodulatory therapy on GIT involvement are scarce. A recently published study reported a beneficial effect of long-term therapy with intravenous immunoglobulins on some of the GI manifestations in patients with overlap of SSc and myositis.^61^ There are no data about the influence on GIT in SSc patients without myositis.

## CONCLUSIONS

The gastrointestinal tract is one of the main systems involved in SSc patients causing significant morbidity and even mortality. There is no single objective measure to assess the extent and severity of GI involvement in SSc patients. A multidisciplinary approach with a rheumatologist, gastroenterologist, and sometimes a nutritionist is mandatory in all patients with severe gastrointestinal involvement. The management of GIT involvement in SSc remains empirical and symptom-driven. Data regarding the influence of immunomodulatory therapy on GIT involvement are scarce. Well-designed and high-powered prospective studies are needed to determine the effect of immunosuppressive treatment on the onset of GI tract disease, especially in early SSc.

## Abbreviations

APC: argon plasma coagulation
GAVE: gastric antral vascular ectasia
GERD: gastroesophageal reflux disease
GI: gastrointestinal
GIT: gastrointestinal tract
LES: lower esophageal sphincter
PCI: pneumatosis cystoides intestinalis
SIBO: small intestinal bacterial overgrowth
SSc: systemic sclerosis
VCE: video capsule endoscopy
